# Protease recognition sites in Bet v 1a are cryptic, explaining its slow processing relevant to its allergenicity

**DOI:** 10.1038/srep12707

**Published:** 2015-08-03

**Authors:** Regina Freier, Elfriede Dall, Hans Brandstetter

**Affiliations:** 1 Department of Molecular Biology, University of Salzburg, Billrothstr. 11, A-5020 Salzburg, Austria

## Abstract

Despite a high similarity with homologous protein families, only few proteins trigger an allergic immune response with characteristic T_H_2 polarization. This puzzling observation is illustrated by the major birch pollen allergen Bet v 1a and its hypoallergenic protein isoforms, e.g., Bet v 1d. Given the key role of proteolytic processing in antigen presentation and T cell polarization, we investigated the recognition of Bet v 1 isoforms by the relevant protease cathepsin S. We found that at moderately acidic pH values Bet v 1a bound to cathepsin S with significantly lower affinity and was more slowly cleaved than its hypoallergenic isoform Bet v 1d. Only at pH values ≤4.5 the known proteolytic cleavage sites in Bet v 1a became accessible, resulting in a strong increase in affinity towards cathepsin S. Antigen processing and class II MHC loading occurs at moderately acidic compartments where processing of Bet v 1a and Bet v 1d differs distinctly. This difference translates into low and high density class II MHC loading and subsequently in T_H_2 and T_H_1 polarization, respectively.

Allergic reactions to proteins of the pathogenesis-related protein family number 10 (PR-10) are mainly related to birch pollen. Additionally more than 70% of patients have pollen-related food allergy^1^. The cross-reactive plant proteins have up to 75% sequence similarity, and all known structures of these cross-reactive proteins share a highly conserved fold^2^. Nevertheless only Bet v 1, the major allergen from birch pollen, is considered to have the capacity to trigger the initial allergic response^3^,^4^. The comparison between isolated and recombinant Bet v 1 led to the discovery of thirteen different isoforms (1.0101 to 1.3001, formerly a-n) with more than 95% sequence identity. Mass spectrometry revealed that Bet v 1a (1.0101) represents at least 50% of the total mass of pollen Bet v 1^5^ while Bet v 1d (1.0401) is present with approximately 10%^6^. Despite the high sequential and structural identity, allergen isoforms can differ drastically in their allergenic potential. Only Bet v 1a induces high IgE antibody production, while Bet v 1d triggers IgG4 expression. Consequently Bet v 1a acts as the sensitizing allergen whereas the hypoallergenic Bet v 1d mainly induces a protective immune response^7^. This might be a general feature of allergens, as hypoallergenic variants were identified in several species^8^. The prerequisite for IgE production is the polarization of naïve T cells to T_H_2 cells, which subsequently release cytokines to stimulate IgE production in B cells. It was shown that antigen density on the surface of antigen presenting cells (APCs) is one of the important factors that influence the fate of a naïve CD4^+^ T cell. High concentrations of class II MHC loaded with antigen-derived peptides promote T_H_1-like responses, whereas a lower density supports T_H_2 cell differentiation^9^. Antigenic peptides are generated by endolysosomal proteases, mostly papain-like cysteine cathepsins S, L, B and V, but also by aspartate cathepsins D and E^10^. Cathepsin L is mostly found in the thymus where it plays important roles in the antigen processing and presentation with relevance to tolerance induction, but to a lesser extent in dendritic cells (DCs). In contrast, cathepsin S is predominantly expressed in professional APCs, namely DCs and B cells^11^. Using activity based probes it was shown that antigens are selectively targeted to cathepsin S in DCs, and are only poorly recognized by other cathepsins^12^. Moreover, in a comparative study of cathepsin B, C, L and S cathepsin L was found to be most effective under lysosomal conditions, albeit also active under endosomal conditions^13^. By contrast, cathepsin S activity was shown to be much stronger in the endosome than in the lysosome^14^. This suggests that cathepsin S is the major protease in early recognition and relevant for the cleavage of antigens. Indeed, the majority of Bet v 1 antigenic peptides, which were produced by extracts of endolysosomal proteases, could be generated by cathepsin S alone^15^.

We therefore used Bet v 1a and Bet v 1d as sensitizing and hypoallergenic model allergens to study differences in the recognition by cathepsin S. The major aim of this study was to find out why hypoallergenic isoforms differ drastically in their immune response, although they have almost identical sequences. Structural dynamics and flexibility can be different between highly similar isoforms, which cannot be derived from crystal structures^16^. In this work we wanted to analyze if structural flexibility is a key difference between hypoallergenic and sensitizing allergens.

## Results

### Early Bet v 1 cleavage sites are not accessible to proteases

**Figure 1:**
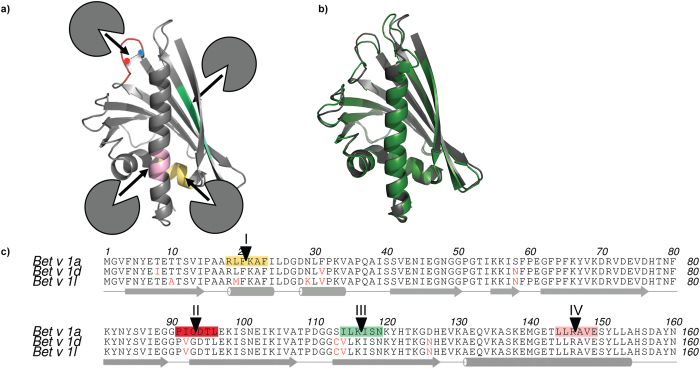
Bet v 1 cleavage sites are cryptic and require reordering for protease access. (**a) Initial recognition sites are harboured within secondary structures.** The initial recognition sites^15^ were mapped into the crystal structure of Bet v 1a (PDB: 4A88). Cleavage sites are indicated with black arrows. The sites are embedded within α-helices (sites I, IV), a 1–4 tight turn (site II), or a β-sheet (site III) **(b) Crystal structures of sensitizing Bet v 1a (PDB 4A88, dark grey) and hypoallergenic Bet v 1l (PDB 1FM4**^32^**, green) share a highly conserved fold.** Cathepsin S recognition sites are located in stabilized regions. The structure-based alignment was performed with topmatch^33^ (**c) Differences in sequence between Bet v 1a and Bet v 1d (red letters) do not coincide with cathepsin S cleavage sites.** The secondary structure is indicated below each row. Coloured backgrounds represent the protease recognition sites from the non-primed site P3 to the primed site P3’. The scissile bond is marked by a black arrow. The alignment was created with ClustralW^34^ and modified with Aline^35^.

The early endosomal cleavage sites in Bet v 1a have been identified and replicated in an *in vitro* cathepsin S cleavage assay^15^. Surprisingly, detailed analysis of the Bet v 1a structure revealed that none of them is easily accessible to the active site of proteases, as they are mostly located in secondary structure elements (Fig. 1a). Additionally, crystal structures of hypoallergenic and sensitizing Bet v 1 isoforms are remarkably similar (Fig. 1b). This counterintuitive observation prompted us to investigate whether and to which extent cathepsin S could recognize Bet v 1a and whether the comparison of the cathepsin S—Bet v 1d recognition could reveal a molecular link to their different allergenic properties.

### Binding of Bet v 1 isoforms to immobilized cathepsin S revealed low affinity sites in Bet v 1a, but high affinity sites in Bet v 1d

**Figure 2:**
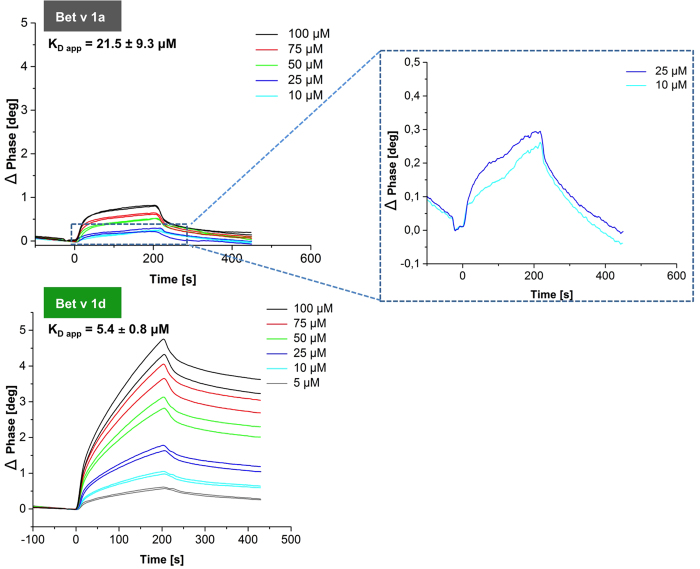
Binding affinity of cathepsin S is four times higher towards Bet v 1d than Bet v 1a. *In trans* activated C25A-cathepsin S was immobilized on a sam®5BLUE biosensor chip. Its binding to Bet v 1a and Bet v 1d was measured at pH 6.7 in a concentration range from 10 to 100 μM. The 4–5 fold increased phase shift in Bet v 1d as compared to Bet v 1a reflects their differences in binding. K_D app_ for Bet v 1a and Bet v 1d were calculated to be 21.5 ± 9.3 μM and 5.4 ± 0.8 μM respectively. The sensograms of 2 independent measurements are shown for Bet v 1a (top) and Bet v 1d (bottom) binding. The calculated dissociation constant K_D app_ represents an average affinity of initial Bet v 1 recognition sites (I to IV). Correspondingly, the enlarged view shows biphasic binding for Bet v 1a at low concentrations.

To study the binding of allergen isoforms to cathepsin S, the C25A inactivated protease was immobilized on a sam5 chip. Its structural integrity was confirmed by high affinity cystatin C binding, an endogenous inhibitor of cathepsin S (Fig. S1). The overall affinity of the hypoallergenic isoform Bet v 1d to cathepsin S was approximately four times higher compared to the sensitizing allergen Bet v 1a (Fig. 2). This finding suggested to us that the substrate recognition sites are hardly accessible in Bet v 1a, consistent with our structural analysis (Fig. 1a). By contrast, the recognition sites must be dynamically more accessible in Bet v 1d.

**Figure 3:**
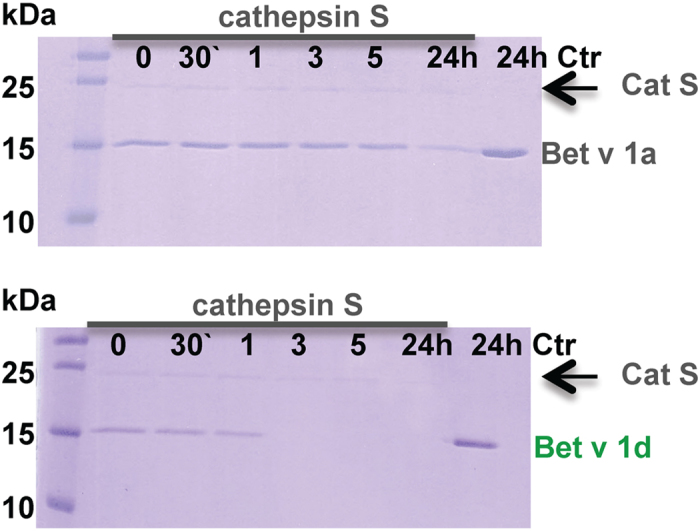
Degradation of Bet v 1a by active wild type cathepsin S was significantly slower as compared to more rapidly digested Bet v 1d. A ratio of 1:10 protease to substrate was incubated at 37 °C at pH 5. Samples were taken after 0, 0.5, 1, 3, 5 and 24 h. In a control experiment Bet v 1 was incubated for 24 h at 37 °C at pH 5 without addition of cathepsin S. Bet v 1d degradation after 1h corresponded to Bet v 1a degradation after 24 h.

To test this interpretation, we compared the degradation kinetics of Bet v 1a and Bet v 1d by active wild type cathepsin S. We found that the Bet v 1a degradation was significantly retarded as compared to more rapidly digested Bet v 1d (Fig. 3), reflecting their differences in binding to cathepsin S.

As illustrated in Fig. 1, Bet v 1 is processed by cathepsin S at several distinct sites. Therefore the here measured affinities should represent an average of the initial recognition sites. Consistent herewith, the binding sensograms typically feature a biphasic association curve with a relatively sharp initial increase in binding followed by a shallow secondary phase. This observation is suggesting that different Bet v 1 recognition sites differ in their affinity towards cathepsin S.

Bet v 1a and Bet v 1d share a high sequence identity of ~95%, i.e. their amino acid sequences differ only at 7 positions. Notably, these point mutations do not overlap with the initial cathepsin S recognition sites, implying that they are at structurally different positions on Bet v 1 (Fig. 1c). Also the crystal structures of hypoallergenic and sensitizing isoforms are virtually identical (Fig. 1b). Therefore, the observed differences in binding affinities and cleavage kinetics of the two Bet v 1 isoforms cannot be explained by the comparison of their sequence or crystal structures.

We therefore postulated that both Bet v 1 isoforms undergo conformational transitions preceding or upon complex formation with cathepsin S, rendering the cleavage sites accessible for proteolysis. The observed differences in cathepsin S binding and processing could then be explained by the higher or lower energetic barrier that needs to be overcome by Bet v 1a or Bet v 1d, respectively. Indeed, minimal changes in the amino acid sequence can have a significant impact on fold stability and flexibility^17^.

### Differences in the fold flexibility of Bet v 1 isoforms relate to their different affinities towards cathepsin S

**Figure 4:**
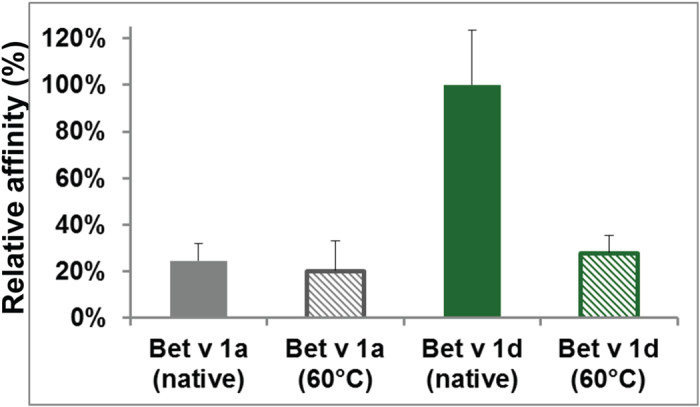
Convergence of binding affinities upon structural destabilization. Whereas Bet v 1a maintained its binding affinity towards cathepsin S, the affinity of Bet v 1d significantly decreased upon thermal destabilization, resulting in an effective convergence of the binding affinities of both isoforms. Binding affinities to immobilized cathepsin S were measured at pH 6.7. Affinities of heat-treated Bet v 1a and Bet v 1d were measured after spin filtration with the same concentrations. Bars represent the mean affinity of 5 measurements; errors show standard deviations.

To test the relevance of the substrates’ fold stability and flexibility for cathepsin S binding, we compared the binding affinities of native and thermally destabilized Bet v 1 molecules. To obtain structurally destabilized Bet v 1, the protein was shortly incubated at 60 °C, close to its melting point of T_m_ = 64 °C^18^. Only soluble, monodisperse fractions of thermally destabilized Bet v 1 were used for cathepsin S binding experiments. We found that the affinities of the Bet v 1 isoforms to cathepsin S converged under destabilizing conditions (Fig. 4). This convergence is due to a five-fold reduction in affinity of Bet v 1d. This change in affinities indicates that the substrate’s fold is indeed important for recognition by the enzyme cathepsin S. The fold encodes an isoform-specific protein dynamics that is critical for the higher affinity of Bet v 1d than that of Bet v 1a.

### Acidic pH is necessary for Bet v 1a, but not Bet v 1d recognition

**Figure 5:**
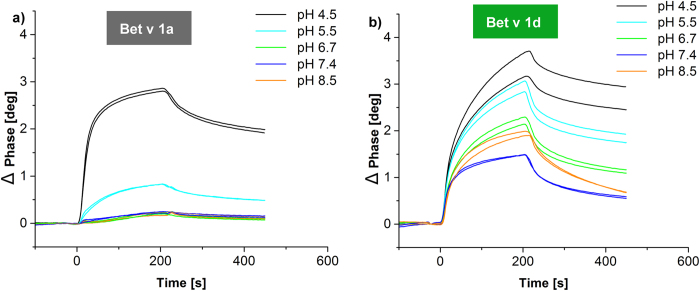
Binding to cathepsin S is pH-dependent and differs significantly between Bet v 1 isoforms. (**a**) Binding of Bet v 1a (10 μM) to immobilized cathepsin S was weak at near neutral pH values (8.5, 7.4 and 6.7). Only at acidic pH (pH ≤ 5.5) binding affinity increased significantly. (**b**) By contrast, binding affinity of Bet v 1d (10 μM) to cathepsin S increased continuously with decreasing pH, except for pH 7.4 where weakest binding was observed. Results for 2 independent measurements are shown.

Since the fold stability of a protein is dependent on several factors such as pH we next analyzed the effect of different pH values on cathepsin S–Bet v 1 complex formation. This factor was especially interesting, since pH is important in the maturation of the endosome. Interestingly, we found that Bet v 1a binding to cathepsin S is pH dependent: pH ≤ 4.5 resulted in a significant increase in binding as compared to neutral pH. Importantly, the recognition of Bet v 1a by cathepsin S at neutral and slightly acidic pH was very low (Fig. 5a). As shown previously, the Bet v 1a affinity towards cathepsin S depended on its fold and the encoded dynamics. Therefore, we conclude that the fold encoded dynamics of Bet v 1a is similarly pH dependent. In contrast, Bet v 1d was bound already at neutral pH, with steadily, but less pronounced increase in binding with lowering pH (Fig. 5b). Furthermore, the binding curves of Bet v 1a and Bet v 1d converge at acidic pH values, indicating a comparable dynamics of both isoforms.

## Discussion

Hyperallergenic and hypoallergenic isoforms of the birch pollen allergen Bet v 1 spotlight a key problem in allergology, namely the causal linkage of molecular properties with their sensitizing potential. In the current study we found low affinity binding of Bet v 1a, but high affinity binding of Bet v 1d, to cathepsin S; the latter protease is critical in its antigen processing^15^. In line with these findings, cathepsin S processed Bet v 1a significantly more slowly than Bet v 1d. Given their high sequential and structural similarity, these findings are intriguing and could be explained by differences in the dynamics of the Bet v 1 isoforms. Importantly, acidic pH ≤ 4.5 triggered the conformational changes that resulted in significantly faster binding to cathepsin S, which was then comparable to Bet v 1d. The underlying molecular mechanism most likely is a better accessibility of the Bet v 1a recognition sites due to increased structural flexibility at low pH.

**Figure 6:**
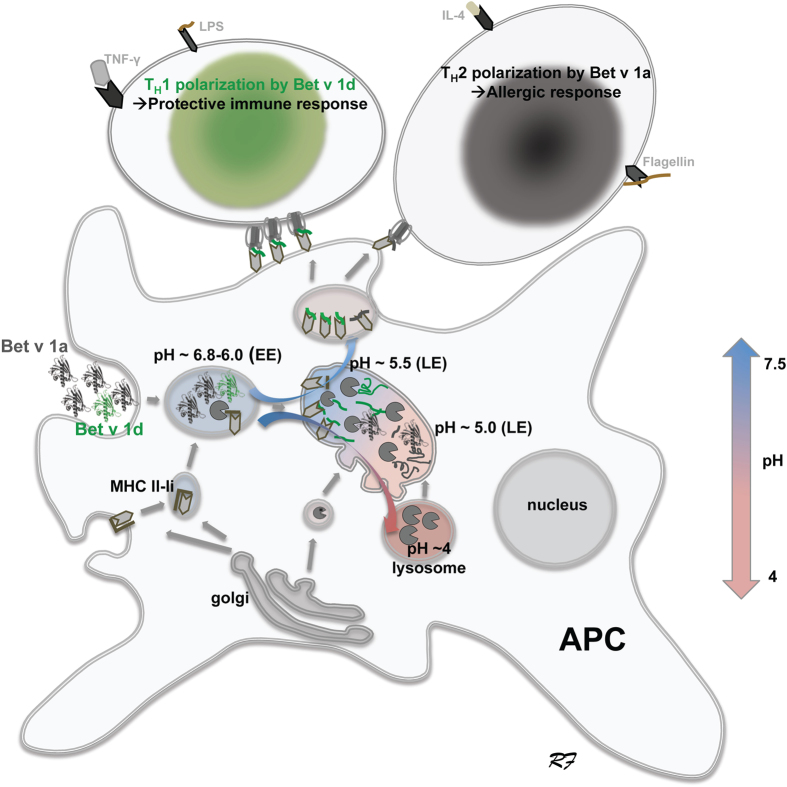
pH-dependent proteolytic resistance of Bet v 1a and 1d selects for the protein degradation and antigen presentation pathway, respectively. Bet v 1a mostly escapes the antigen presentation pathway, because its cleavage sites are cryptic at pH ≥ 5.5; therefore the majority of Bet v 1a ends up in the degradation pathway. By contrast, Bet v 1d is readily processed in the antigen presentation pathway with pH ≥ 5.5. Consequently, large amounts of Bet v 1d-derived peptides will be loaded and presented on class II MHC, explaining the protective T_H_1 response. The large amounts of endocytosed Bet v 1a allergen together with its low proteolytic processing at pH ≥ 5.5 warrant a continuous supply of Bet v 1a peptides for presentation albeit at low concentration, thus explaining its allergic T_H_2 response. pH values for the early endosome (EE, pH 6.8-5.5), the late endosome (LE, pH 5.5-5) and the lysosome (pH 5-4) are approximate values as reported in the literature^21^,^36^,^37^.

The pH value is a key factor in endosomal processing and endocytosed proteins experience an increased acidification from pH 7 to pH 4 during endosomal maturation. APCs have developed strategies to prevent the rapid acidification of endosomal compartments. This allows proteins to remain intact for a longer period of time, which is a prerequisite for the presentation pathway of antigens^19^,^20^ (Fig. 6). Consequently a continuous supply of intact protein allows persistent generation of peptides suitable for presentation (>12 aa). Our results show that the non-sensitizing Bet v 1d will be preferentially processed in the late endosome (LE) at slightly acidic pH (≥5.5^21^), resulting in antigenic peptides for class II MHC presentation; by contrast, only few antigenic peptides will result from Bet v 1a at this milieu. Cathepsin S is, unlike many other lysosomal cysteine proteases, stable and active under a broad pH range, including the class II MHC presentation compartment^22^. Indeed relatively high levels of cathepsin S activity were detected in the early endosomes (EEs) of antigen presenting cells, especially of dendritic cells, less in macrophages^23^. Class II MHC is synthesized and assembled in the endoplasmatic reticulum (ER), directed via the invariant chain li either directly to EEs or more commonly to the plasma membrane, from where it is internalized again by endocytosis^24^. Newly synthesized class II MHC-li complexes are processed mainly by cathepsin S, before peptide loading (reviewed in^25^). Of similar importance, class II MHC loaded complexes reside primarily in late endosomes (LEs)^26^. By contrast, mature lysosomes at more acidic pH values contain only little class II MHC and are unlikely to generate any functional peptide loaded complex^27^.

In conclusion, hypoallergenic variants such as Bet v 1d are largely processed within the LE by cathepsin S, with a preferential class II MHC loading with Bet v 1d-derived peptides. The resulting high density of Bet v 1d-mediated synapses of APCs with naïve T-cells induces their polarization to T_H_1 cells with a protective immune response. In contrast, the Bet v 1a-mediated APC—T cell synapses are sparse and consequently induce the polarization of T cells into T_H_2 with an allergic immune response. Large amounts of endocytosed Bet v 1a are necessary to maintain the continuous supply of loaded class II MHC complexes at low dose, critical for T_H_2 polarization^28^. The majority of Bet v 1a protein will be completely recycled along the degradation pathway in the LE and the lysosome at acidic conditions (pH ≤ 4.5; Fig. 6). Finally, the here provided concept of pH-dependent proteolytic resistance of allergen offers new treatment options for allergic patients. The *in vitro* screening for and identification of orally available low molecular weight compounds that expose the cryptic proteolytic recognition sites in allergens have the potential to induce an immune protection, similar like specific immune therapy.

## Methods

### Cloning, expression, and purification of cathepsin S

Human procathepsin S cDNA clone BC002642 was obtained from GeneCopoeia (Rockville, US). For subcloning of expression constructs *Escherichia coli* strain XL2 Blue (Stratagene, La Jolla, USA) was used. To obtain glycosylated protein, procathepsin S was expressed in the *Leishmania tarentolae* system (LEXSY; Jena Bioscience, Germany). The encoding DNA was amplified by polymerase chain reaction (Eppendorf Mastercycler ep gradient thermal cycler) with human cathepsin S full-length cDNA clone BC002642 as template and primers containing an *Xba*I restriction site and six codons for histidine (CCTCTCTAGAGCACCACCATCACCACCACGTGGCACAGTTGCATAAAGATCCTA CCCTG) and a *Not*I restriction site (GAGGGCGGCCGCTCACTAGATTTCTGGGTAAG). The PCR product was cloned into the pLEXSY-sat2 vector using the *Xba*I and *Not*I restriction sites. Point mutations C25A and S21C were introduced with ‘Round-the-horn’ site-directed mutagenesis^29^. All expression constructs contain an N-terminal signal sequence for secretory expression, followed by an N-terminal *His*_6-_tag for purification, which remains with the propeptide after autoactivation. The identity of expression constructs was confirmed by DNA sequencing. Stable transfection of expression constructs into the LEXSY P10 host strain was achieved by electroporation, and subsequent selection of positive clones was performed via addition of nourseothricin (Jena Bioscience). Cells were grown at 26 °C in BHI medium (Jena Bioscience) supplemented with 5 μg/ml hemin, 50 units/ml penicillin and 50 μg/ml streptomycin (Carl Roth). Large-scale expression was carried out in 500 ml shaking flasks at 26 °C until OD600≈3 was reached. Recombinant procathepsin S was purified from the LEXSY supernatant via Ni–NTA superflow resin (Qiagen, Hilden, Germany). Eluates in 400 mM NaCl, 20 mM Tris-HCl pH 8, 300–500 mM imidazole were concentrated using Amicon Ultra centrifugal filter units (3 kDa molecular-weight cutoff, Millipore). For long-term storage of procathepsin S the buffer was changed to 20 mM Tris pH 8, 20 mM NaCl, 5 mM DTT using NAP-5 desalting columns (GE Healthcare). For autoactivation wild-type procathepsin S was incubated in a buffer composed of 5 mM EDTA 2.5 mM DTT and 100 mM sodium acetate pH 4.0 for up to 24 h at 37 °C. To activate the C25A active site dead mutant, the buffer was set to 100 mM NaCl, 5 mM EDTA, 10 mM sodium acetate pH 5, and human legumain produced as described in^30^ was added at a ratio of ≈1:500, and incubated at 30 °C for at least 2 h. To remove uncleaved procathepsin S and the non-covalently bound prodomain, the pH of the buffer was raised to pH 8, and the samples were again applied to Ni-NTA columns. The flow through contained mature cathepsin S.

### Expression and purification of Bet v 1a and 1d

Recombinant Bet v 1a and Bet v 1d were expressed in *Escherichia coli* strain BL21(DE3) as non-classical inclusion bodies. The expression construct was cloned into a modified pET-28b vector, lacking the N-terminal *His*_6_-tag. Cells were grown in 600 ml LB medium supplemented with 20 μg/ml kanamycin at 37 °C to an OD_600_ of 1.0. Expression was induced with 1 mM IPTG, and cells were harvested after 4 h. Purification of the non-tagged Bet v 1 was performed with acidic salt precipitation, hydrophobic interaction (phenyl-sepharose) and anion exchange (diethylaminoethano–sepharose) chromatography as previously published^31^. Additionally size exclusion chromatography was applied as a final purification step, using a Superdex75 column (GE Healthcare). Purified Bet v 1 was stored in 20 mM imidazole, pH 7.4, and 50 mM NaCl at −80 °C.

### Proteolytic processing assay

Bet v 1a and Bet v 1d (c = 0.25 mg/ml) were dialyzed against a buffer composed of 100 mM NaCl, 5 mM EDTA, 2 mM DTT and 10 mM sodium acetate pH 5, and spin-filtrated before protease digestion. 120 μl of Bet v 1 were mixed with 25 μl activated cathepsin S (0.1 mg/ml in 100 mM NaCl, 5 mM EDTA, 2 mM DTT and 10 mM sodium acetate pH 5), and incubated at 37 °C. This corresponds to a ratio of 1:10 protease to substrate. 20 μl samples were taken after 0, 0.5, 1, 3, 5 and 24 h. In a control experiment Bet v 1 supplemented with 25 μl buffer without addition of cathepsin S was incubated for 24 h at 37 °C.

### Interaction studies using SAW-technology (Surface Acoustic Waves)

The sam®5BLUE biosensor instrument (nanotemper, Munich, Germany) was used to test the interaction of different Bet v 1 isoforms with cathepsin S. *In trans* activated C25A-cathepsin S was coupled to the surface of a sam short-chain COOH sensor chip. The protein was incubated in a buffer composed of 20 mM sodium acetate pH 5.0, 100 mM NaCl, 1 mM EDTA and 0.5 mM DTT. 240 μl of a 500 nM cathepsin S solution were injected to the chip, which was activated with a 1:1 mixture of 400 mM 1-[3-(dimethylamino)propyl]-3-ethylcarbodiimide hydrochloride (EDC) and 100 mM N-hydroxysuccinimide (NHS). Sequential injection of increasing ligand concentrations (Bet v 1 isoforms) were performed to calculate the affinity constant K_D_. Freshly spin-filtered Bet v 1 samples were applied at concentrations of 5 μM to 100 μM in a buffer composed of 20 mM imidazole pH 6.7, 50 mM NaCl. For the measurement of heat-destabilized Bet v 1a and 1d samples, protein concentrations of 10 to 100 μM and 2.5 to 40 μM were used, respectively. The coated chip was equilibrated in the same buffer. Between each injection residual ligand was removed with regeneration buffer (10 mM citric acid pH 3.0). Experiments were repeated with two individually coated chips. SAW phase changes were recorded and used to calculate the affinities based on pseudo-first order kinetics (k_obs_), from which the apparent dissociation constants K_D app_ were determined by linear regression. Fitmaster, a customized add-on for Origin (OriginLab, Northampton, MA) was used to fit the raw data. Best fitting was obtained when an incomplete regeneration (uncoupled k_diss_) was chosen as mathematical model. To test the effect of pH on Bet v 1 binding 10 μl of each isoform (a and d) were dialyzed against five different buffers (20 mM Tris-HCl pH 8.5, 20 mM PBS pH 7.4, 20 mM imidazole pH 6.7, 20 mM acetic acid pH 5.5, 20 mM acetic acid pH 4.5, all except PBS were supplemented with 50 mM NaCl). The chip was equilibrated with the respective buffer prior to injection of Bet v 1.

### Induction and control of destabilized Bet v 1

To destabilize the fold, a 100 μM stock solution of Bet v 1 was incubated for 10 min at 60 °C, close to, but lower than the melting point (T_m_ = 64 °C; quantitative precipitation occurred after 15 to 20 min). Potential high molecular weight aggregates were removed by spin filtration. The concentration before and after filtration was controlled by measuring the absorption at 280 nm wavelength. To test monodispersity of heat-treated Bet v 1, 70 μL of a 100 μM stock solution were compared with untreated Bet v 1 samples by dynamic light scattering (Fig. S2). Only monomeric Bet v 1 was used to study the binding to cathepsin S.

## Additional Information

**How to cite this article**: Freier, R. *et al.* Protease recognition sites in Bet v 1a are cryptic, explaining its slow processing relevant to its allergenicity. *Sci. Rep.* **5**, 12707; doi: 10.1038/srep12707 (2015).
